# Medico-Chirurgical Transactions, Published by the Medical and Chirurgical Society of London

**Published:** 1834-01-01

**Authors:** 


*Medico-Chirurgical Transactions, published by the Medical and Chirurgical Society of London.* Vol. XVIII. Part I.—London, 1833. 8vo. pp. 300.

It has always been a subject of regret with the philosophic physician, that, from the smallness of the sphere of observation allotted to each individual practitioner, many interesting objects of inquiry can hardly be investigated with advantage by any single observer; and we are compelled, therefore, to collect into one focus the scattered rays of light transmitted from various places, and through a long series of years. Yet this method of instruction has its obvious disadvantages: these foreign theories and antique cases seldom strike the mind with the same force as the registered observations of our contemporaries and countrymen: indeed, the unreasonable scepticism of many has risen to so high a pitch, that to them it is as if the hard-working physicians of the sixteenth and seventeenth centuries had never existed; and the remarkable cases recorded by the doctors of the olden time are mentioned only to be doubted. Nothing can be more unphilosophic than this exaggerated scepticism; the honesty of those venerable teachers of physic is sufficiently shown by many similar cases which have occurred in our own days; and, to expect Nature to renew *all* her pathological prodigies for our instruction, is to imitate the absurdity of the French *Marquise*, who, having arrived too late at the observatory, hoped that the astronomer would have sufficient politeness to repeat the eclipse for her entertainment.

It is to palliate these incurable evils, and concentrate as it were in one moment the experience of an age, that medical societies have been formed; but it is to be lamented that their researches have rarely been directed to elucidate any *single* point, so that the advantage derived from these combinations of talent and learning has been that of discussing a subject rather than simultaneously investigating it. In the volume, however, which is at present before us, it happens, rather, we should think, by accident than design, that there are three papers on the same subject, namely, on fatty discharges from the bowels. The first, which is by Dr. Bright, is entitled "*Cases and Observations connected with Disease of the Pancreas and Duodenum.*" We will give the commencement of the first case in the words of Dr. Benjamin Babington, who tells it exceedingly well.

"James Barnes, aged forty-nine, clerk in a waggon office, a man of sober and regular habits, began to complain, in March 1827, of immoderate thirst and appetite, with a constant pain in his loins. He made water very frequently, and his urine was of a pale colour. These symptoms, accompanied by emaciation, increasing upon him, he applied for advice successively to several medical men. It was not, however, until the middle of August following that his disorder was pronounced to be diabetes mellitus. In the beginning of September he first became affected with jaundice, but his change of colour was not preceded by any painful sensations. Under different dispensaries and practitioners he was subjected to various medical treatment, until the 4th of December, the period at which I first saw him. He was then reduced to a state of great debility, although still by no means a thin man. His thirst and appetite were constant, his pulse was eighty and soft, his skin deeply tinged with bile. His urine, of which he made about nine pints in twenty-four hours, was deep coloured, and left a yellow stain on linen; it was very sweet, and had a specific gravity of 1039. His evacuations by the bowels were copious and light coloured. The liver was at this time but obscurely felt through the integuments, as these were still filled out with adipose matter. My directions were, that the patient should live upon animal food, with a moderate portion of greens or lettuces, and that he should eat as little bread as possible, and avoid all kinds of roots, fruits, and such articles of diet as contain sugar or meal; that he should drink tea without sugar, and at his dinner stale porter, or port-wine and water. With his diet thus regulated, a bitter aromatic mixture, with subcarbonate of soda, was prescribed." (P. 3.)

The diabetes now gradually disappeared, and, with a view of removing the bilious obstruction, the extract of taraxacum was prescribed, first with, and afterwards without, blue pill.

The diabetes indeed was now cured, but a new, and perhaps even more alarming, disease took its place.

"On Friday the 28th, without any notable change of symptoms, the patient began to pass a quantity of yellowish fatty matter, much resembling butter that had been melted and had become again solid. This matter followed the fæces, and, as it was evacuated in a melting state, it was perceived on the surface of the dejection. We could not trace the origin of this change to any thing that had been eaten; for he had been upon a rigid system of diet from the time that he had been under my care, and had taken nothing of a fatty nature since that period. Meat, indeed, he had used in abundance, but his wife had constantly and carefully removed the fatty parts. For the subcarbonate of soda in the tonic mixture, liquor potassæ, in the proportion of fifteen minims to the dose, was substituted to meet more effectually this symptom." (P. 6.)

The liquor potassa did away the oiliness of the motions for a few days; but the patient continued to sink, and in another week the stools again contained fatty matter. As slight diarrhœa existed, a chalk mixture was ordered, with aromatic confection and tincture of opium. The diarrhœa was checked, but the patient became progressively worse, and died on the 1st of March. The following were the post-mortem appearances:

"*Sectio Cadaveris.* The limbs were remarkably flaccid; the whole skin was of a dark yellow colour; there was great general emaciation, and the legs were slightly œdematous.

"Both the pleura costalis and pulmonalis were tinged with bile. The lungs were healthy in structure throughout, except the lower lobe on the left side, where very acute pleuritis had recently occurred, throwing out a thick coating of gelatinous fibrin, which broke down readily when the lungs were drawn forwards. This was partly deposited on the lower surface against the diaphragm, and partly on the posterior part of the ribs near their angle, and it was highly tinged with bile. The lung itself was implicated, and a small abscess, of the size of an olive, was formed near the edge, where it rests upon the diaphragm. The pericardium was deeply stained with bile, and the large vessels near the heart were quite yellow, this colour pervading their whole thickness. The heart was rather small and contracted, but retained its natural proportions, and the valves were healthy. The abdomen contained rather more than a gallon of dark olive-coloured fluid. The gall-bladder, distended with very dark bile, was seen with its fundus projecting when the parietes were first removed. The liver was of a dark olive colour, from the bile with which it was pervaded. The ducts were greatly enlarged. The common duct was large enough to admit the little finger freely when passed from above downwards, and its internal surface presented a honeycomb or reticulated appearance, and terminated by a cul de sac in the diseased substance of the pancreas, and at its shut end a rough white deposit had taken place, probably either of fibrin or cholesterine.

"The *head of the pancreas* formed, with some of the surrounding glands, a hard globular mass, round which the duodenum turned, and to which both it and the pylorus were firmly joined; and in two parts, where the pancreas and duodenum were welded together by the disease, ulcers of a hard and schirrous character had taken place, penetrating the whole thickness of the intestine; one of them of the size of a shilling, and the other not larger than a silver penny-piece. The pancreas was hard and cartilaginous to the touch, and of a bright yellow colour.

"A section of the liver looked like a fine-grained dark greenstone porphyry, or very dark Aberdeen granite; the ducts, which were throughout enlarged, being completely filled with bile which flowed from the incision. In different parts of the liver a few irregular masses occurred, of a firm hard consistence, but shaded off into the substance of the liver, and not bearing the appearance of circumscribed tubera. The stomach was slightly vascular. The spleen natural in structure, but its external surface mottled with cartilaginous deposit. The intestines were tolerably natural, but somewhat opake, and the internal lining rather pale. The kidneys were to external appearance perfectly healthy; but the tubular parts shewed themselves more plainly than usual, when the kidney was torn open, and in some of the tubes were white specks from a deposit either of fibrin or of calculous matter. The pelvis of the kidney was not vascular, but was tinged with bile. The lining membrane of the bladder was remarkably healthy, and free from all vascularity; but its netlike appearance bespoke more than usual action in the muscular coat. The aorta and common iliacs were in many patches diseased with bony deposits surrounded by dark spots, where the internal surface had been destroyed by ulceration or absorption." (P. 10.)

The subject of the second case, a woman aged fifty, was admitted into Guy's Hospital, under Dr. Bright's care, November 19th, 1828. She was then suffering from jaundice and occasionally violent pain in the lower part of the abdomen; a few days afterwards greasy matter was observed in her alvine evacuations: it was at first taken to be undigested castor oil, but was found to remain when the supposed cause was discontinued. This patient soon quitted the hospital, and returned to Gravesend, her usual place of residence, where she died on the 16th of February. Dr. Bright predicted that the post-mortem appearances would be similar to those observed in the case of Barnes, and the following extract will show that he was not mistaken:

"*Sectio Cadaveris, Feb.* 18, 1829. Skin generally of a deep yellow colour, varying in parts to greenish brown, not very unlike the dark colour of the Creole. General emaciation, but by no means to the extent sometimes seen: indeed, on the abdomen there was a considerable portion of fat of a deep yellow colour.

"The lungs were in a very healthy state, except that they were both bound firmly at the posterior part by very strong adhesive bands, and the whole surface was tinged moderately with bile. Heart healthy, but small, and not firm.

"On opening the abdomen, the omentum rather loaded with fat. No peritoneal disease or adhesion, except on the superior surface of the liver, which was attached in several parts by long adhesions to the diaphragm.

"The cause of pressure on the bile-ducts was immediately obvious; for, on placing the hand near the pylorus, a hard lump, of the size of a common egg, was easily felt, and was soon discovered to be the head of the pancreas itself, and not the glands surrounding that part, forming a yellow mass like the boiled udder of a cow, almost cartilaginous. Its texture was uniformly hard and unyielding, and the whole pancreas partook of the same, but in a less degree. The head of the pancreas was firmly and inseparably glued to the duodenum, and the hardness very nearly surrounded that viscus. Laying open the duodenum, its internal surface was uneven and ulcerated, the ulcer having eroded the whole of the coats; and in the portion lying on the head of the pancreas it was of a soft consistence and light yellow colour, communicating with the substance of the tumour, here irregularly softened or suppurating to the extent of a small chesnut. In the midst of the ulcer a little nipple-like body was seen projecting on its surface, which proved to be the orifice of the common duct of the gall bladder. This was still pervious, as the thick bile could be squeezed out of the gall bladder through it. But it was obvious that this had either lately become pervious by the ulceration of its orifice, or the ulceration of the hard mass in which it was imbedded, or that its situation in the contracted duodenum had acted as a compressing cause; for the gall bladder was distended, containing at least four ounces of thick dark green bile, which stained the lining membrane of the deepest colour. The gall bladder, though thus loaded, was not tense, and conveyed the idea of a somewhat flaccid bag, so that I should have pronounced it to have been more distended lately than at the present moment. The disease of the mucous coat of the intestines occupied the outside of the ridge forming the pylorus, which was strongly marked, but was not schirrous; yet, on passing the finger before the pylorus was cut open, the hardened neighbouring structure produced all the effect of a stricture.

"The liver was of its natural size, containing several round tubera sprinkled through various parts, from the size of a grain of rice to that of a nutmeg. These were not very numerous, but five or six were seen on the superior surface, where they were perfectly circular, and a little depressed in the centre. They were decidedly harder than the surrounding liver, but did not separate freely from it; on the contrary, generally seemed to be shaded off into the surrounding parts, and the texture of the liver was discernible in them. The larger tubera were soft and yellow in the centre. The general structure of the liver was healthy, rather soft, and of a dark olive-green colour. The biliary ducts were enormously distended; their branches near the margins of the liver were visible on the surface, and they were filled with a fluid watery bile.

"The mucous membrane of the stomach was rather spongy, of a reddish tint, and it contained half a pint of brown grumous matter, apparently secreted from its surface. We examined several portions of the mucous membrane, both of the small and large intestines, but they presented nothing peculiar except a rather spongy texture, and on some parts a grey colour. The spleen was healthy, but soft. The kidneys large and flaccid, tinged with bile throughout, particularly their lining membrane. The large vessels appeared healthy, and the lumbar and other glands were not diseased. The bladder contained some yellow urine. The uterus was rather thick and round in its form, the cavity large, and the glands at the mouth of that organ put on the appearance of vesicles at first sight. They were distended with glairy, almost gelatinous mucus, of a yellow colour, which could with some force be squeezed from their orifices." (P. 17.)

In the third case, the patient, a young woman of twenty-one, entered Guy's Hospital, labouring under jaundice and anasarca. The fatty excretion, though well marked, was far less abundant than in the previous cases, as it amounted only to a pellicle on the surface of the evacuations. The patient died in six days; and the following was the result of the anatomical examination.

"*Sectio Cadaveris.* The whole body was decidedly tinged with bile; some yellow serum escaped from the abdomen when it was opened. The liver was immediately seen considerably enlarged from distension, and of a dark olive-green colour. The fundus of the gall bladder projected; it was distended with bile of a dark-green colour. The quantity which it contained could not have been less than four ounces, and the ducts were as large as the little finger till they entered the duodenum, where the orifice was very small, and it required considerable force to make the bile pass into the intestines. The whole of the intestines were somewhat distended, and in several places, when viewed externally, their puckered and discoloured appearance plainly indicated that mischief had been going on within; and, at one part of the small intestines, an actual perforation, large enough to admit the point of the little finger, had taken place, but a slight adhesion, which gave way in the examination, prevented the feculent matter from being effused.

"The whole course of the intestines was now laid open, and fungoid excrescences and ulcerations were found distributed at irregular intervals, from their commencement at the pylorus to the termination at the colon. Whether any existed in the rectum, I am not quite certain. These ulcers might be traced throughout their whole progress. They began by small elevations, generally upon the edges of the convolutions, of a light yellow or white substance; and those which had arrived at the size of a pea generally had a depression in the centre, as if from partial ulceration. The depressed part was softer than the surrounding edge, and, if the tumour was squeezed, a whitish puriform or cerebriform matter issued from pores upon its surface. This, however, was better seen when the whole disc had increased to the size of a sixpence. About this time, or sometimes sooner, the surface lost its light and clean appearance, and became covered, sometimes with a sloughy mass, but more frequently with a dark grumous coat, apparently from blood which had exuded, and become changed on its surface. The mass was now elevated nearly half an inch, the edges inverted or cup-shaped, and the centre either raised with the loose fungoid slough and blood, or, if this had come away, was deeply excavated, going on in its progress to perforate the substance. In two instances these fungoid ulcerations communicated immediately with large fungoid excrescences, probably glands situate externally to the intestine. One of these was close to the ileo-colic valve, where the external ulcer was black with grumous exudation, and formed the mouth of a cavity which would admit the finger into a mass involving the glands of the mesocolon. Another of the same kind, but less completely opening into the external diseased mass, occurred in a portion of the duodenum.

"The mesenteric glands were involved in this disease; and the renal capsules, but more particularly the left, had suffered from the same affection.

"The kidneys were healthy. The uterus was also healthy, but its appendages had suffered great irritation. One of the fimbriated extremities was completely bound down, and the orifice obliterated; and the ovaries were corrugated, and contained vesicles in different states of disease.

"In the liver no fungoid disease shewed itself, but its texture was natural, though gorged with bile.

"The pancreas was most deeply involved in the disease. It formed a hard mass near its head, and then, a more healthy portion intervening, another hardened mass was seen near to the spleen, when another small portion remained healthy at its termination; so that it might be said to be occupied by two fungoid tubercles, which involved two thirds of its whole structure. The limits of these diseased masses were not distinctly defined, but they were of a more yellow colour than the rest of the organ, and destroyed the lobular structure of the gland.

"The spleen was unusually small.

"In the chest, the same disease was found affecting the bronchial glands, and, in the form of one round fungoid tubercle of the size of a moderate plum, in the apex of one of the lungs: this was imbedded completely in the substance, and was of a yellow white colour." (P. 22.)

In all these cases, as Dr. Bright observes, there was chronic disease terminating in jaundice, which again was followed by fatty dejections. "In the result of examination after death, we have likewise some circumstances which exactly coincide in all: *obstructed biliary ducts; the liver gorged with bile; fungoid disease attacking the head of the pancreas; and malignant ulceration on the surface of the duodenum.*" (P. 26.) Our author was inclined to attribute these singular evacuations to the disease of the pancreas; and accordingly, when consulted in two cases in which pancreatic disease was suspected, but in which the stools were not fatty, he withheld his assent from the diagnosis. In the first of these consultations the patient was a coachman, who was wasting away under obscure organic disease, "and a hardness was perceptible at the pit of the stomach before he died. On examination after death, tumours of a schirrous character were found in the liver, in Glisson's capsule, and at the small curvature of the stomach; but the pancreas was perfectly healthy." (P. 29.)

Our author's negative diagnosis was equally fortunate in the other instance.

"The second case was that of a man, aged fifty, who had suffered deep-seated pain at the scrobiculus cordis for several months, together with occasional palpitation of the heart, and abdominal pulsation. He wasted much, and lost his colour, but no tumour nor aneurismal enlargement could be felt. It was evident that the heart was diseased. No direct proof could be obtained of abdominal disease; and, as nothing of the peculiar fatty appearance could be discovered in the alvine evacuations, I refused to assent to the opinion which had been entertained of the existence of pancreatic obstruction. About a month after I saw this man he gradually sunk, becoming slightly jaundiced a day or two before his death, having also had cough, with mucous expectoration.

"*Sectio Cadaveris.* Very general and old adhesions were found between the pleura costalis and the pleura pulmonalis; some parts of the lung were hepatized from old disease, while other parts were emphysematous, and some recent irritation was observable in the bronchial membrane. The heart adhered very closely to every part of the pericardium, and was enlarged in its substance universally. The mitral valves, and the semilunar valves of the aorta, were slightly diseased.

"The liver contained a good deal of blood, which was distributed irregularly between the acini, so as to give a mottled or nutmeg appearance. The acini were light coloured, a little tinged with bile, the gall bladder was full of bile, but not distended beyond its natural size. The ducts were pervious; but it was with some difficulty we could make the bile pass from the gall bladder to the duodenum, apparently owing to its tenacious condition. The pancreas was perfectly healthy, nor was there any material derangement in the other abdominal viscera." (P. 29.)

We are then presented with three cases in which these oleaginous stools did not exist, although the pancreas was diseased; and again, Dr. Bright mentions instances in which fatty stools existed without any evidence of diseased pancreas, the patient not sinking under the disease. Hence this obscure and interesting subject is still open to further investigation. Dr. Bright concludes his paper with the following judicious remarks:

"There is but one further observation which I would make in connexion with the cases which have been related. All of those in which the oily evacuation has been observed, have been cases of decided malignant, and (as far as the pancreas is concerned) we might perhaps say, schirrous disease. Now, it is a fact which I have observed in several cases, that the bile is very apt to undergo that change which leads to the deposit of concretions of adipocire in the gallbladder, in patients labouring under schirrus, (as females with schirrous mammæ, for instance,) where the disease either has or has not attacked internal organs; and I think it arises as a fair question, therefore, whether the peculiar appearances of the alimentary discharges may not depend on the same disposition, be it what it may, which leads to this unnatural deposit in the gall bladder; and, should this prove to be the case, the symptom would be diagnostic of the nature of the diseased action rather than of its seat." (P. 56.)

The next paper is entitled "*Cases of Jaundice, with Discharge of Fatty Matter from the Bowels, and a Contracted State of the Duodenum.*" Mr. Lloyd narrates the case of a patient who expired after an illness of ten months, during the last two of which he frequently passed fatty matter by stool. The stomach was found to be enormously enlarged, and its coats thickened. We extract an account of some of the other appearances, in the words of our author.

"Immediately beneath the pylorus a hard tumour was discovered, which proved to be principally made up of a portion of the duodenum, the head of the pancreas, some absorbent glands, and condensed cellular substance. The duodenum, towards its middle, including that part into which the ductus communis choledochus enters, was so contracted, that its cavity was in great part obliterated; so much so, that, till separated from its adhesions, it would very little more than admit the larger end of a small blowpipe to pass through it. The greater disease was at the posterior part of the intestine, the part connected with the head of the pancreas. In no other portion of the alimentary canal was any appearance of disease detected. The pancreas was healthy, except at that part more immediately connected with the duodenum, where it had undergone some slight degree of induration, as if it had been inflamed. Its duct, at the termination in the duodenum, was completely obstructed: in the rest of its course it was not only pervious, but it was larger than natural, and contained a brownish fluid, of rather a yellowish tint, resembling in some respect the fatty matter in the state that it was when it passed from the intestine." (P. 64.)

Fluctuation had been felt during life when the liver was pressed upon, which was accounted for by the following morbid alterations:

"An incision being made in the liver, several ounces of dark thick bile instantly gushed out, as if a bag of bile had been cut into. But, on examination, it proved that the bile had been contained in enlarged pori biliarii, some of which were so large that they readily admitted the forefinger. They existed in an enlarged size, more or less, in every part of the liver, proceeding from near its surface, and directly passing to the hepatic duct; so that there was the freest communication between the bile in them and the contents of the gall bladder." (P. 65.)

There was no malignant disease in any part of the body.

Dr. ELLIOTSON is the last, but not the least writer on this subject; for his essay is distinguished by his usual vivacity and learning. Our author prefaces the account of the cases which he offers to the society with an historical sketch of some of those already recorded.

"I need not remind the society, that ambergris, or properly grey amber, is a fatty substance, consisting chiefly of something called ambreine, and analogous to cholesterine; or that it is supposed to be produced by disease in the alimentary canal of the spermaceti whale (*Physeter macrocephalus*,) from which it is frequently discharged, and is found either floating near the coast or lying on the shore of India, Africa, and Brazil, though sometimes discovered in the animal after death, occasioned by its accumulation, or the state which gives rise to it. Some declare that it is never seen higher than six or seven feet from the anus; and a mass amounting to 182 pounds has been found in the animal.*

" Fatty matters, which have an external origin, are occasionally discharged from the human alimentary canal. Castor oil is frequently seen liquid in the evacuations. Riverius states that he saw fatty substances passed from the intestines in one case, after a large quantity of fat had been eaten, or had been taken in broths; and in another after a large quantity of oil had been swallowed. Other instances of fatty discharges from the stomach or intestines, liquid or in lumps of various sizes, some unquestionably, and others possibly, derived from external sources, are related in the German Ephemerides.

" Some suppose that olive-oil may concrete with mucus when taken into the alimentary canal, and thus explain certain cases of this description.

" Old authors, however, detail instances of fatty discharges from the intestines that do not appear to have originated externally; and, of every variety of those old cases, I can adduce a modern and indisputable example.

" In some instances the fat was discharged solid.

" 1. Mœllenbroccus relates that a man, at Halle, where he practised, discharged from the bowels, for two years, a large quantity of fatty substance, (*materiem pinguem*,) not unlike the fat of beef, grew thin and weak, and died tympanitic.

" 2. Mœbius mentions a similar daily discharge of a substance exactly like human fat, (*materiem humanæ pinguedini plane similem*,) from a woman, who wasted away.

" 3. 'A pious and virtuous matron,' about fifty years of age, and residing at Duisbourg, suffered some years from a pain at the stomach, that was relieved by nothing, and at length became much worse; when one day the pain extended all over the abdomen with extreme severity, and she discharged above three pounds of fat. From that moment she speedily and perfectly recovered. The fat was white, very pure, and in detached pieces, surrounded with pellicles. It was not mixed with the fæces, had no smell, and was preserved by the woman for many years. Fabricius Hildanus paid a visit, he says, on the 6th of August, 1612, to Dr. Daniel Daniel, the most eminent physician of Duisbourg, who was well acquainted with the matron, and had seen the fat. Daniel gave Hildanus a feast worthy of Lucullus, (*necnon Lucullico convivio ab ipso exceptus essem*,) and completed his hospitality by sending for the woman to tell her own story: *ut et ego matronam ipsam viderem, et quæ acciderant, ex proprio ore exciperem, annotaremve*." (P. 67.)

After several similar instances, Dr. Elliotson gives one

* Phil. Trans. 1783.

from Tulpius, in which the fat was discharged in a liquid state.

" 'Alithea Epicornia, a slender delicate woman, who had been frequently indisposed, either from tertian ague or obstruction of the spleen, discharged, at length *every day*, for above fourteen months, a large quantity of *yellow fat*, which *lay upon* the fæces, like melted butter; and sufficient, had it been collected, to have filled a number of vessels, (*plurimum flavescentis adipis; incumbentis stercori, instar butyri liquefacti. Sed eâ plerumque copiâ, ut potuisset, modò quis illum collegisset, replere aliquot vascula.)* When thrown into the fire, *it burnt with a bright flame;* and, after the fæces on which it lay had cooled, *it concreted to the consistence of rather solid fat.* But, what was very remarkable, there were neither tormina, emaciation, nor even colliquative fever.' Sixteen years afterwards she was in excellent health." (P. 74.)

In a case treated by Dr. Elliotson himself, the patient laboured under phthisis, diabetes mellitus, diarrhœa, and violent pain in the abdomen, and in the dorsal portion of the spine. A quantity of yellow substance, like a concrete oil, was observed in his stools; when put into the fire, it burnt with a large flame, and Drs. Prout and Faraday were satisfied of its oily nature. "On examination after death, all the intestines looked yellow and greasy, as though they had been soaked in oil. Numerous black points were seen in some parts of their mucous membrane, the same that are frequently noticed after fever and chronic diarrhœa; but no other morbid appearance existed in the alimentary canal. The liver was healthy, and the gall bladder full of thick dark bile. The pancreatic duct, and the larger lateral branches, were crammed with white calculi. The kidneys were sound. The lungs were tuberculated and ulcerated." (P. 78.)

In another case, under the care of Mr. Pearson, of Clapham, both fat and oil were passed. In another patient, again, attended by Dr. Prout, fat was voided from the intestines, but mixed with blood and other things. "After death, the cæcum was found much thickened, and its mucous membrane, as well as a considerable portion of the mucous membrane of the colon, ulcerated. All the other abdominal viscera were perfectly healthy." (P. 79.)

Tulpius, however, goes beyond this:

" 'But what do we say of Margaret Appelmania, an innkeeper, who, in her seventieth year, discharged precisely similar fat from both the intestines and the bladder, and likewise without fever, emaciation, or colliquative excretion.' 'Towards the close of the disease, however, she did become feverish, and in consequence so emaciated, that death found her little else than a juiceless, driedup corpse: *cujus æstu, anile hocce corpusculum adeo emarcuit, ut mors in ipsa, vix quicquam repererit, præter exsuccum, ac aridum cadaver.*'"

Even this pathological portent has occurred again; the patient, who was seventy-nine years old, was attended by Mr. Pearson. No post-mortem examination was allowed.

We will conclude our extracts from these interesting papers with Dr. Elliotson's brief notice of the treatment of these cases:

"In regard to treatment, the lady mentioned by Dr. Babington was always relieved almost at once by a few ounces of olive oil, and Dr. Simpson appears to have cured two cases by the exhibition of an immense dose of it. In imitation of this practice, I gave my patient two ounces of olive oil for two successive days, and four ounces on the third, which, however, he made two doses of, with the effect of vomiting and purging; and he certainly from that time discharged much less of the oily matter, and suffered much less pain in the abdomen and back. But the disease, I fear, lies as open to enquiry, both in its pathology and its treatment, as the analogous disease of diabetes." (P. 84.)

It would be superfluous to offer any comment on these very remarkable cases. Though the number is small, the post-mortem appearances have been so various as to baffle all attempt at referring the symptoms to any one structural alteration; and the treatment has been so unsuccessful, that, should another such case arise, we have neither practice nor theory to guide us.

We now turn to another subject. Mr. ARNOTT has enriched the "Transactions" with a clear and well-written account of a case in which he performed the operation of Œsophagotomy. The gullet of the patient, who was only two and a quarter years old, was obstructed by a fragment of bone, which ultimately "proved to be the spinous process of one of the dorsal vertebræ of a sheep." After many ineffectual attempts to remove the bone, the operation was performed, "almost four weeks after the accident," says Mr. Arnott; but, in fact, five weeks and a day, if his dates are correct. The operation seems to have been very skilfully performed, but, though anatomically perfect, it was therapeutically useless, as the child died fifty-six hours afterwards. "The right lung, with the exception of its upper part, was hepatized, and portions of it, thrown into water, sunk. Sections of it presented a mottled gray appearance and granulated texture, with here and there a drop of yellow matter from the extremity of the bronchial tube. In a less degree, and more partially, hepatization was observed in the left lung." (P. 92.) Mr. Arnott thinks that œsophagotomy is not so very formidable an operation as it is generally supposed to be, and mentions two instances in which it has lately been performed with success in France.

Mr. Cæsar Hawkins has contributed an interesting paper on "*Cases of Sloughing Abscess connected with the Liver, with some Remarks on Encysted Tumours of that Organ.*" It would be impossible to give even an analysis of this essay, as it is exceedingly long, and somewhat discursive; we shall therefore content ourselves with referring our readers to it, assuring them that it will well repay the trouble of perusal.

We next come to a similar subject, namely, a case of *Aqueous Encysted Tumour oj the Kidney;* for which also we are indebted to Mr. Hawkins: this paper may be considered in the light of an appendix to the former one. Mr. Macilwain gives an account of two cases of *Deep-seated Nævi, successfully treated by the introduction of Setons;* and Dr. Elliotson has furnished some additional facts respecting *Glanders in the Human Subject.*

Mr. Key's essay *on the Ulcerative Process in Joints* is sensible and judicious, but contains nothing particularly worthy of being extracted.

Mr. Langstaff gives a "*History of a Case of Medullary Sarcoma, which affected several important Viscera.*" One fact is very singular. "About twelve hours prior to the patient's dissolution, uterine hemorrhage commenced, which was succeeded by the expulsion of a fœtus, of the third or fourth month of utero-gestation, and which was in a putrid condition: the placenta was not expelled." Dissection shewed that the natural structure of the ovaria had entirely disappeared; and it may therefore very naturally be asked, how could impregnation be effected? Mr. Langstaff answers this by supposing that the perfect disorganization of the ovaries may not have taken place until after the impregnation.

The following case is so remarkable that we make no apology for extracting the whole:

"A young man in his eighteenth year fell from the top of a loaded cart upon his right hip, the injury of which was attended by the following symptoms. He was wholly unable to move the limb, and suffered severe pain when it was moved by another person. The thigh was bent to a right angle with the pelvis, and could not by any means be extended. Abduction of the thigh was difficult. The limb was everted, at first slightly, afterwards in a greater degree. The soft parts around the hip joint were considerably swollen. There was no shortening of the limb, but rather the appearance of a lengthening of it in the erect posture, probably from the obliquity in the position of the pelvis. No crepitus could be felt in any movement of the limb.

"The foregoing symptoms were not considered to indicate conclusively the existence either of dislocation or fracture. The age of the patient was unfavorable to the occurrence of a fracture of the neck of the thigh bone, the general opinion therefore of the several surgeons to whose judgment the case was submitted favouring the belief of a dislocation into the foramen ovale, forcible extension of the limbs was made by means of the pulleys, and the thigh then moved in several directions, by which the head of the bone might be replaced in its socket.

"About two months after the accident, the patient was received into St. Bartholomew's Hospital. His health was now found to be much deranged. His pulse was frequent and hard. He complained of pain in the head, also in the injured hip, and down the opposite thigh. This illness was considered to be the effect of cold, but it did not yield to the treatment which was adopted. He remained nearly in the same state for about a month, and during this period, on account of the derangement of the health, no examination was made of the injured hip. At length, eruptions appeared generally over his body, which were considered to be small-pox, and in two days afterwards he died.

"In the examination of the body, no other morbid appearances were discovered besides those of the injured hip joint. The capsule of the joint was entire, but a little thickened. The ligamentum teres was uninjured. A line of fracture extended obliquely through the neck of the femur, and entirely within the capsule. The neck of the bone was shortened, and its head, in consequence, approximated to the trochanter major. The fractured surfaces were in the closest apposition, and finally united nearly in their whole extent by bone. There was an irregular deposition of bone upon the neck of the femur, beneath its synovial and periosteal covering along the line of the fracture.

"The foregoing case is remarkable from the occurrence of a fracture of the neck of the femur within the capsule at an early age, and it is, I believe, the only example of it on record. In the memoirs of the Academy of Surgery, Sabatier has related the case of a boy aged fifteen, in whom, after a fall upon the hip, lameness ensued, and sometime afterwards, a shortening of the limb to the extent of three inches, with a projection of the trochanter major, and an inclination of the whole limb inwards. The patient recovered sufficiently well to be able to walk, but with a considerable restraint in the movements of the thigh. Here it may be presumed a fracture had occurred, but it is certain that the seat of it could not have been within the capsule of the hip joint from the great extent of the shortening of the limb.

"It will be remarked that in the instance now recorded, notwithstanding the free and repeated examinations of the limb, and the forcible extension of it by the pulleys; in short, with every circumstance, except the age of the patient, unfavorable for a bony union of the fracture, this had been nearly completed. If this case had occurred at an advanced period of life, we may be certain that there would have been but a very imperfect union of the fracture, and it shews satisfactorily, that in the ordinary cases of fracture of the neck of the femur within the capsule, the age of the patient, and consequent deficiency of vascular action, especially in the separated head of the bone, is the most influential of the causes to which the failure of a bony union has been in general ascribed." (P. 256.)

Mr. Stanley, the contributor of this case, has also given an *Essay on Irritation of the Spinal Cord and its Nerves, in connexion with Disease in the Kidneys.* He narrates several cases in which the patients exhibited the usual symptoms of spinal disease, but, on post-mortem examination, not the spine, but the kidneys were diseased. It is remarkable, however, that he does not mention whether the urine was alkaline in any one of these patients.

This portion of the "Transactions" concludes with a paper by Dr. Sims on *Malignant Tumours, connected with the Heart and Lungs,* of which he has seen several instances.

It is impossible to terminate our notice of this volume without expressing our admiration of the professional knowledge displayed in it: yet we cannot but wish that in some of the longer papers the authors had favoured us with a *resumé* of the principles which it was their intention to defend or illustrate. Such an enunciation of medical theorems is useful, not only as a guide to the reader, but a check to the writer; it is not merely the cynosure of students' eyes, but the star by whose propitious aid the adventurous author steers clearly over the unfathomable ocean of cases, treatments, and dissections.

